# Solvent Effect to the Uniformity of Surfactant-Free Salmon-DNA Thin Films

**DOI:** 10.3390/polym13101606

**Published:** 2021-05-16

**Authors:** Jake Richter, Moses Nnaji, Heungman Park

**Affiliations:** Department of Physics and Astronomy, Texas A&M University-Commerce, Commerce, TX 75428, USA; jrichter2@leomail.tamuc.edu (J.R.); mnnaji95@gmail.com (M.N.)

**Keywords:** DNA films, spin coating, film uniformity, solvent effect

## Abstract

Fabrication of surfactant-modified DNA thin films with high uniformity, specifically DNA–CTMA, has been well considered via drop-casting and spin-coating techniques. However, the fabrication of thin films with pure DNA has not been sufficiently studied. We characterize the uniformity of thin films from aqueous salmon DNA solutions mixed with ethanol, methanol, isopropanol, and acetone. Measurements of thickness and macroscopic uniformity are made via a focused-beam ellipsometer. We discuss important parameters for optimum uniformity and note what the effects of solvent modifications are. We find that methanol- and ethanol-added solutions provide optimal fabrication methods, which more consistently produce high degrees of uniformity with film thickness ranging from 20 to 200 nm adjusted by DNA concentration and the physical parameters of spin-coating methods.

## 1. Introduction

Deoxyribonucleic acid (DNA) has always been an interest in the biological sciences but has recently become an interest to researchers in materials science, physics, and chemistry for applications as a biodegradable component in optical and electronic devices [1,2,3,4,5,6]. Specifically, the band gap of DNA makes it favorable to function as a hole injection/electron blocking layer in organic-based electronic devices [7,8,9]. Previous authors have demonstrated the ability of DNA to improve LED performance [7,8,9,10,11,12,13] and as a component in the fabrication of photovoltaics [14,15,16,17]. Moreover, DNA films can be used as a matrix and doped with functional materials that can alter the optical and electronic properties of the thin film, which may be useful for different types of organic devices [18,19,20]. Due to the discovery of these promising physical properties, DNA thin-film devices have become a notable research interest in materials science.

Despite the potential of DNA-based thin-film devices, not much study has been reported to systematize the fabrication of DNA thin films from aqueous and alcohol solutions [14]. Previous research has focused on thin films fabricated from surfactant modifications, primarily with cetyltrimethylammonium (CTMA) modified DNA thin films with consistent results [13,15,16,17,21,22,23,24,25]. Thus, research has focused on controlling the electronic and optical properties of surfactant-modified thin films, and applications have relied on characterizing results from DNA–CTMA complexes. Recently, groups have been making progress towards efficient surfactant-free thin films because the toxicity of CTMA makes DNA–CTMA solutions ineligible for many biological applications and becomes insoluble in water [14,19,26]. However, research on surfactant-free thin DNA films has focused on how to affect the physical properties of the films, such as optical dispersion and refractive indices [19,25,27,28,29,30] and not on improving the efficiency of thin-film fabrication or standardizing the production of films with tunable thickness within a controlled range of uniformity. Developing a process for unmodified DNA is important for consistent research results and enabling future researchers to develop their own thin films with or without modification.

We comment on some complications in the current methodologies for reporting thin-film fabrication. First, there are varying reports on how solutions are prepared. It has been noted that sonication times of pure DNA solutions will affect the size of polymer chains and, as a result, will affect both the viscosity and characteristics of the resulting thin film [24]. This, along with other significant variations in the solution preparation phase, such as mixing practices and minor changes in the deposition phase of spin coating, can cause serious issues when reproducing thin films, especially when solutions are spin-coated onto a hydrophobic surface. For viscous solutions, the type of dispense (i.e., static or dynamic dispense) can have considerable effects if there are bubbles in the solution before or after deposition, and the area of initial deposition can have a tremendous impact on the resulting uniformity of the film even when one controls for other parameters [31,32,33,34,35]. Additionally, previous results rely on microscopic measurements of uniformity using AFM and SEM techniques, although the usefulness of these measurements are undeniable at the smallest scales, there is little evidence of expectations for macroscopic uniformity or thickness. Thus, it is plausible that researchers can be fabricating films promising microscopic measurements, but the existence of uniformity over millimeter scales could compromise the results of the film and its applications.

We present a systematic analysis of spin-coating procedures to fabricate DNA thin films from solvent added aqueous solutions with tunable thickness and desired macroscopic uniformity (millimeters to centimeters). Since there are a continuous number of changes that may be made to the fabrication process, such as DNA concentration, solvent/water ratio, spin rate, and so on, we focus on a range of DNA concentrations and alcohol choices; we then modify spin rate for each one. We present analysis on quantifying uniformity and it is assumed that a reasonable interpolation of the data between concentration and solvent choice can be made upon data analysis.

## 2. Materials and Methods

### 2.1. Aqueous DNA Solution Preparation with Solvents

Deoxyribonucleic acid salts extracted from salmon testes (salmon DNA salt) was purchased from Sigma-Aldrich. DNA solutions were made by first dissolving a determined mass of salmon DNA in a pre-determined amount of distilled water for concentrations of DNA that were 4 mg/mL (~0.4 wt.%), 8 mg/mL (~0.8 wt.%), or 12 mg/mL (~1.2 wt.%). For consistency, all DNA concentrations were dissolved in 15 mL of water; thus only the total mass of DNA was changed. Once the DNA and water were added to the vial, a quick vigorous shake was given to ensure all DNA salts were integrated into the solution. The aqueous solutions of DNA were stirred at room temperature with magnetic bars for 24 h to develop a homogeneous mixture. Upon completion of the initial mixing process, each solution of DNA was then separated into two smaller vials to be diluted by a chosen solvent with one of two ratios, either 0.5 mL of solvent to 2 mL of DNA solution or 2 mL of solvent to 2 mL of DNA solution. The resulting solvent–water–DNA mixtures were then stirred with magnetic bars for 24 h to ensure proper mixture with the solvents, thus a total mixing time of 48 h. The high-concentration DNA solution at 12 mg/mL became highly viscous like gel.

### 2.2. Thin-Film Fabrication by Spin Coating

Silicon wafers were cut by 1 cm × 1 cm and treated with sonication baths in clean acetone, water, and methanol and dried with nitrogen between each bath. Once the sonication bath of methanol was complete and the wafer dried, it was subject to UV–ozone treatment for 25 min. The thickness of the native oxide of the silicon wafers was measured by an ellipsometer, which was about 1.9 nm. Within 1 h of being cleaned, the wafers were used as a substrate for a thin film. 

A clean wafer was then centered on a spin coater, and thin films were created by depositing the same volume, ~125 μL, of DNA solution onto the center of each wafer until the solution naturally covered the entirety of the wafer (i.e., no forced spreading with a pipette tip), a process which generally requires less than 5 s. For high-viscosity solutions this is imperative since rearrangement of deposited solution with a pipette tip, as is commonly practiced with inks and low-viscosity solutions during static dispense, can lead to unwanted inconsistencies in the development process and create many confounding variables that render experimental analysis impractical. Immediately after dispense the spin process was initiated and lasted for 300 s. This process was repeated on new wafers controlling only for the RPM and solvent-DNA solution. After the film was deposited onto the wafer it was vacuum-dried for 1 h at ~20 mTorr. The films were subsequently measured with an ellipsometer.

### 2.3. Macroscopic Uniformity Measurement by a Focused-Beam Spectroscopic Ellipsometer

**Figure 1:**
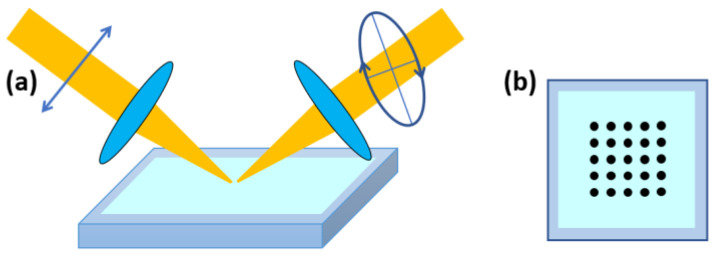
(**a**) A schematic diagram of focused-beam ellipsometry. (**b**) A 5 × 5 measurement grid on DNA films on silicon wafers. The spacing between the measurement points is 1.25 mm.

Ellipsometry measurements were taken on a 5 × 5 grid of points using an alpha-SE Ellipsometer (J.A. Woollam, Lincoln, NE, USA). Each wafer with a DNA thin film was centered on the measuring platform, and measurements were taken at 1.25 mm intervals as shown in Figure 1. The focused-beam size is about 500 μm. A Cauchy model was used in the transparent visible-to-near-IR regions to determine the thickness of the films.

### 2.4. UV–VIS Absorbance Measurements

DNA solutions even at 1 mg/mL produce absorbance peaks that are ill-defined with less than 0.1% transmittance in the expected UV range for DNA for the spectrometer. To solve this issue, a procedure similar to the one discussed Section 2.1 was used to produce solutions of DNA at 0.04 mg/mL (~0.004 wt.%) and diluted with the same volume of solvents to make 1:1 ratio solvent-to-water DNA solutions. The solutions were placed into 1 cm path length fused quartz cuvettes for absorbance measurements. For each measurement, a baseline of solvent–water solution was taken prior to the UV–VIS measurement for solvent–water DNA solution to isolate the DNA absorbance peaks and determine any effect alcohol choice might have on DNA.

## 3. Results

### 3.1. Thin Films by Pure Water–DNA Solutions

Table 1 and Table 2 give values for DNA thin films from pure water–DNA solutions at varying DNA concentrations and spin rates. The standard deviation of a film divided by its average thickness was used to determine a quantitative value for uniformity weighted by the average so that thicker films will not be disadvantaged by a naturally higher standard deviation. Therefore, a lower Std/Ave corresponds to higher uniformity. We observed that pure water–DNA solutions created large sample-to-sample variation, which can be caused by uneven distribution of the solution onto silicon wafers and minor misalignment of the wafers, and relative humidity can be a significant factor in film formation by spin coating [36,37,38]. The two data sets in Table 1 and Table 2 demonstrate that pure aqueous-based solutions of DNA produce inconsistent results particularly at the low RPM. However, we observe in the following sections that methanol and ethanol additions can greatly improve general uniformity and consistency of film formation; and that higher concentrations after dilution with alcohol experienced the greatest improvement in uniformity. This result can be explained by the viscosity change after dilution and faster drying, which affects the ability of DNA to coat evenly on the silicon surface.

### 3.2. Thin Films by Water–Methanol DNA Solutions

**Figure 2:**
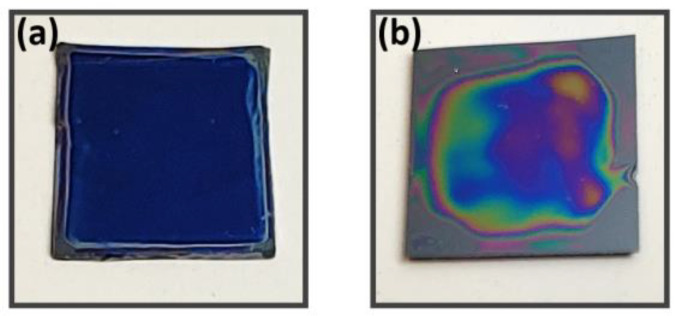
Pictures of two samples: (**a**) 1.2 wt.% + 25% volume of MeOH at 3k RPM with average thickness = 107 nm and Std/Ave = 2.01%; (**b**) 1.2 wt.% + 100% volume of MeOH at 1k RPM with average thickness = 327 nm and Std/Ave = 12%.

Table 3 and Table 4 demonstrate the effects of adding methanol to aqueous DNA solutions as the resulting solvent–DNA mixtures produce films consistent with expected results. Both show a highly consistent result in the high RPMs (3k and 5k). If the film thickness is greater than 80 nm, the film uniformity is visually monitored from the interference color patterns as shown in Figure 2. The inconsistencies of the pure water–DNA films demonstrate the poor ability of pure DNA solutions to make consistent results. Despite this, it is possible to have a film made from pure DNA solutions with good thickness and uniformity, but any perturbation from optimal conditions, such as small misalignments, inconsistent UV–ozone treatment, or other factors, will significantly affect the final film. Solvent-added solutions demonstrate an ability to overcome such perturbing.

### 3.3. Thin Films by Water–Ethanol, Isopropanol, 1-Butanol, and Acetone DNA Solutions

Table 5 confirms observations from above discussion. We expect that the most significant effect of alcohol choice on the final film will be at the 1:1 ratio of solvent to aqueous DNA. Thus, focusing on that ratio, we may conclude simple relations between alcohol choice and optimal conditions for thin-film fabrication.

We also note the importance of the time change between solution deposition and initiating the spin coating. Since spin coating is dependent on the quality of the initial solution, if there are bubbles within the deposited solution, then the time to remove the bubbles can create inconsistent results. Experiments with additions of acetone to DNA solution and allowing a waiting period between deposition and initial spin, we observed a decrease in the uniformity of the resulting film with only a 40 s wait. It is suspected this was due to the quicker drying rate of the acetone–DNA mixture. While this may be a more intuitive result of the experiment since a long-enough waiting period will result in a drop-casted film. The important observation is that even in a short time frame, the quality of the final film can be greatly affected.

**Figure 3:**
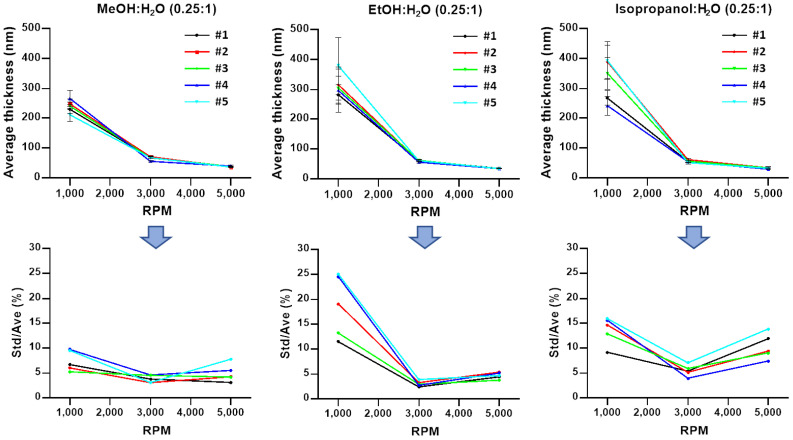
A total of 5 data sets of average thickness and standard deviation from 5 × 5 measurements of 0.25:1 MeOH/H_2_O, 0.25:1 EtOH/H_2_O, and 0.25:1 isopropanol/H_2_O DNA solution films. An amount of 2 mL of aqueous DNA at 8 mg/mL (0.8 wt.%) diluted with 0.5 mL of methanol, ethanol, and isopropanol, respectively.

**Figure 4:**
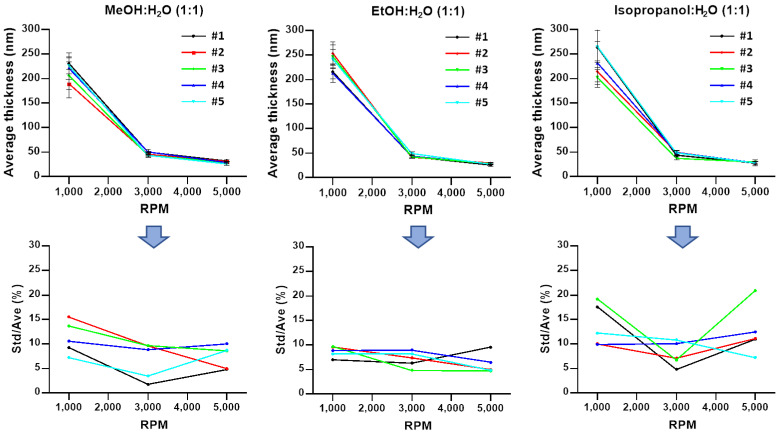
A total of 5 data sets of average thickness and standard deviation from 5 × 5 measurements of 1:1 MeOH/H_2_O, 1:1 EtOH/H_2_O, and 1:1 isopropanol/H_2_O DNA solution films. An amount of 2 mL of aqueous DNA at 8 mg/mL (0.8 wt.%) diluted with 2 mL of methanol, ethanol, and isopropanol, respectively.

Moreover, the problem of predicting uniformity even without a waiting is intractable. This is a result of the plateauing and in some cases a decline in the degree of uniformity as the RPM increases. For example, at 1000 RPM the films are the least uniform and exhibit a significant increase in uniformity at 3000 RPM (lowest ratio of standard deviation to average, Std/Ave). In most cases, at 5000 RPM the uniformity ‘levels off’ or experiences a slight decrease (represented by an uptick in Figure 3 and Figure 4 Std/Ave). This trend is noticeable regardless of DNA concentration; thus, there are non-trivial optimal conditions that determine uniformity. That is, an increase in the RPM will not consistently produce films that are more uniform. Instead, for the solvents studied here, there may be an optimal uniformity that does not occur when the RPM is at its highest. The preceding observation is made based on the choice of defining a film’s uniformity by the ratio of its standard deviation and its average.

Solvent choice has little effect on final film thickness with a noted exception of solutions with 25% added isopropanol and were spun at 1000 RPM. The average thickness is greater than the average thickness produced by either 25% added methanol or ethanol (Figure 3). This appears to be a combination of the natural variability in film-to-film results of isopropanol solutions and the added variability of film-to-film results for highly concentrated solutions of DNA at a lower RPM. For the other series of data presented, in most cases thickness varies by less than 10 nanometers at higher 3000 and 5000 RPMs, suggesting that a high RPM dominates the spin-coating process even for concentrated solutions of DNA. Due to extra dilution, adding 100% solvent results in statistically thinner films at 1000 and 3000 RPMs when compared with an addition of only 25% solvent. At 5000 RPM this trend continues, however, the difference in thickness because of extra solvent dilution is significantly less when compared with thickness differences at 3000 RPM. As the RPM increases, the variability in thickness between films decreases, and thus it becomes significantly easier to predict the expected average thickness.

For 25% alcohol additions, the volume of the original solution, methanol was more consistently uniform than ethanol or isopropanol from a lower 1000 RPM to a higher 5000 RPM. At alcohol additions that were 100% of the original volume, ethanol was more consistently uniform than methanol, which was different from the trend observed at lower additions of methanol. However, in the case of 100% and 25% added solvents, there were individual films of methanol, ethanol, or isopropanol that appeared to be more uniform than the solvent that performs the best on average. This statistical phenomenon is well observed in methanol- and ethanol-added solutions at higher RPMs, where their performance was similar, and at higher spin rates despite the solvent addition, isopropanol produces the most inconsistent and non-uniform films.

Thus, in the case of uniformity, alcohol choice is important. It should also be noted that at 1:1 solvent/DNA ratio with 1-butanol, DNA will dissociate from the original solution, leading to an insufficient solution due to a clumping of large visible chains of DNA and immiscible solvents; this is a case where DNA–CTMA performs well due to the change in polarity. Upon examination of these tables alone, we cannot conclude why DNA performs best under these conditions, but it does motivate further research.

**Figure 5:**
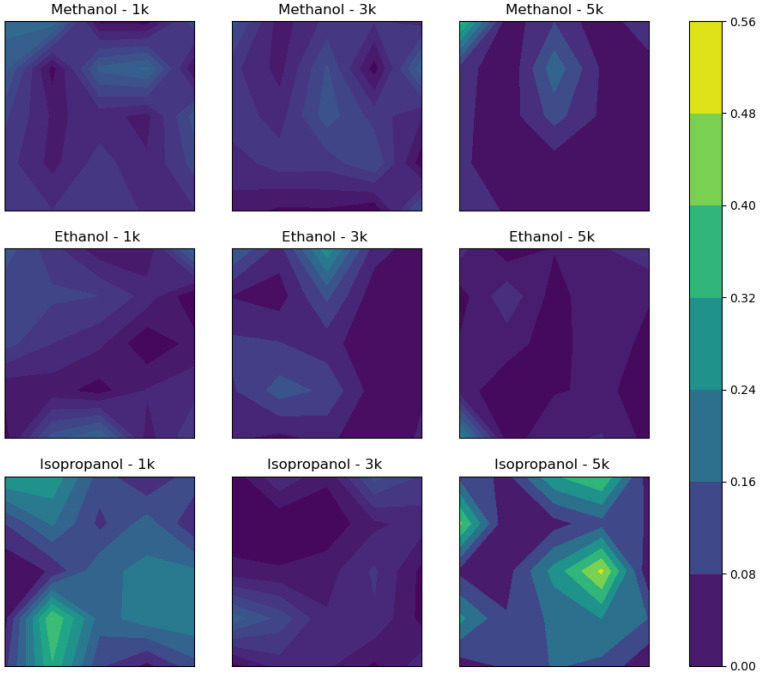
Contour plots constructed from the measurements made on films developed by 100% solvent additions at the specified RPM. A selected data set from Figure 4.

**Figure 6:**
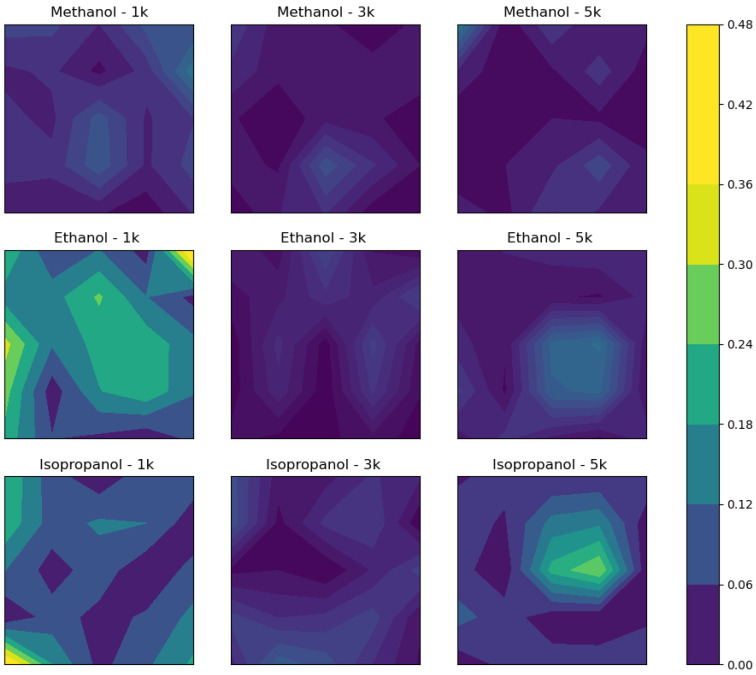
Contour plots constructed from the measurements made on films developed by 25% solvent additions at the specified RPM. A selected data set from Figure 3.

We illustrate uniformity in a visually intuitive manner via contour maps (Figure 5 and Figure 6). The contour maps are constructed from selected data sets of 5 × 5 data points from the ellipsometry measurements of Figure 3 and Figure 4; however, for the purpose of creating the contour maps, each point is modified by subtracting the average film thickness from the thickness at that point, and then the subsequent absolute value of the difference is divided by the average thickness, thus normalizing the points to the average film thickness, which is given by

(1) $$Countour\;map\;point=\frac{\mathrm{thickness}\;\mathrm{at}\;a\;\mathrm{point}\;\mathrm{on}\;\mathrm{film}-\mathrm{average}\;\mathrm{thickness}\;\mathrm{of}\;\mathrm{film}}{\mathrm{average}\;\mathrm{thickness}\;\mathrm{of}\;\mathrm{film}}$$

The modified matrix of points is then mapped as a contour. The results illustrate how uniform the samples are among neighboring points, which provides a more robust understanding of uniformity and avoids complications with describing a sample uniformity using only standard deviation and thickness and has the advantage of utilizing complete information about the geometry of the films from the original data. For example, Figure 5 corresponds to thin films made by a one-to-one ratio of DNA solution-to-solvent addition (100% addition), and isopropanol at all RPMs exhibits random distributions of large variations in thickness occurring at the edges and center of the samples, whereas methanol- and ethanol-added solutions appear to have more centralized points of discontinuity and exhibit less significant continuity changes from point to point. Figure 6 demonstrates a similar pattern for 25% solvent additions, where again methanol and ethanol have centralized regions where thickness is significantly different from the average.

### 3.4. UV–VIS Measurements

**Figure 7:**
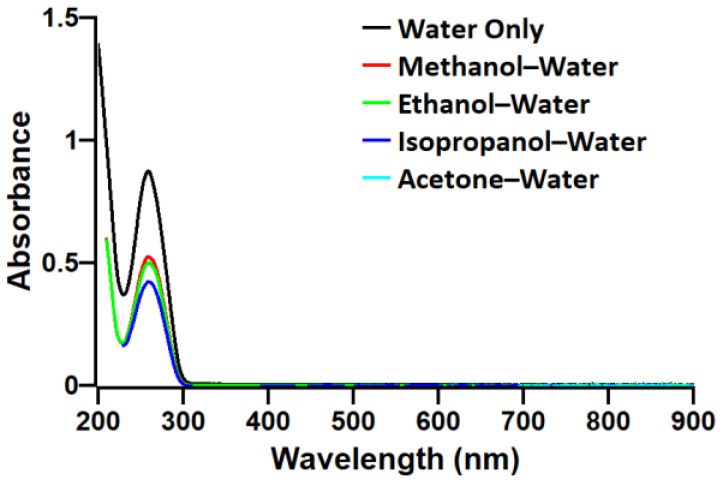
UV–VIS absorbance spectra of DNA solutions with/without solvents. Acetone–water DNA solution does not show the characteristic absorbance peak at 260 nm due to 330 nm UV cutoff of acetone. The optical path length of the cuvettes is 10 mm.

**Figure 8:**
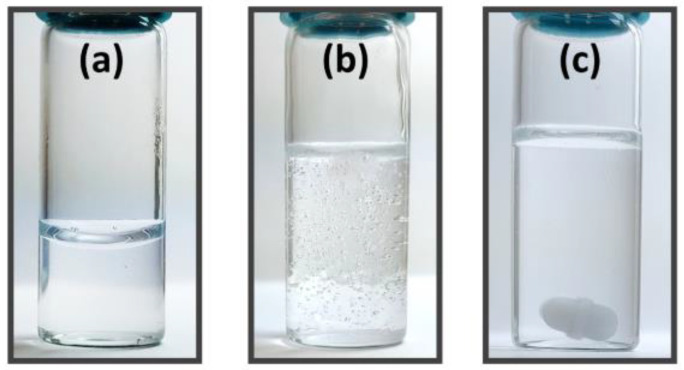
Pictures: (**a**) A highly homogeneous aqueous DNA solution. (**b**) The same volume of isopropanol is added into the sample of (**a**) with a vigorous quick shake. Bubbles in the entire solution and coalesced DNA at the top are present. (**c**) After 1 h of stirring of the sample of (**b**) with a magnetic stir bar, the DNA was dissolved homogeneously in isopropanol–water mixture.

UV–VIS analysis indicates that there is no significant absorbance peak shift in DNA regardless of the existence or choice of solvent (Figure 7). This is expected from previous research [20,30,39]; however, methanol, ethanol, and isopropanol exhibit different magnitudes in the peaks of DNA, and acetone solution is not graphed here since it exhibits a hard UV cutoff where the expected DNA peak is. Methanol and ethanol produce peaks of similar magnitude, while isopropanol produces a noticeably lower peak. This is also observed in cases where the DNA concentration is higher (data are not shown). We note that it is common for adding solvents to aqueous-based DNA solutions, which will lead to contraction in total volume, compared with the entire solution being purely aqueous, in which the contraction will lead to an increase in absorbance since there ought to be a denser spatial distribution of DNA in solvent-added solutions. However, DNA solutions interact differently with the alcohols, which may partially be explained by the polarity of the solvents, while all of the solvents (methanol, ethanol, isopropanol, acetone) are miscible in water. The polarity matches the magnitude of the curves, with methanol being the most polar and isopropanol being the least. This would similarly explain lab observations that when isopropanol is added to solutions with DNA, the DNA is visibly dissociated and coalesced in water and isopropanol mixture, but after magnetic stirring, the DNA aggregates become stable and are homogeneous within the solution by eye as shown in Figure 8. Due to the original coalescence, it is suspected that the DNA polymers may be greatly affected, and UV–VIS measurements indicate that the number of concentrations is lower despite volume contractions from adding non-aqueous solvents, thus resulting in solutions that are less dense in the number of DNA particles in the solutions.

## 4. Summary

Salmon DNA was dissolved in water, mixed, and later diluted with solvents of methanol, ethanol, isopropanol, acetone, and 1-butanol. The solutions were then used to make thin films by spin coating. The efficacy of the solvent additions to make uniform films was tested by varying DNA concentration, solvents, and spin rates, which are the major parameters of spin coating. Thickness measurements were made on a 5 × 5 grid of points on the substrate via focused-beam ellipsometry to determine an average and standard deviation, and subsequently quantify the uniformity. UV–VIS analysis was also performed on the solutions to demonstrate any changes in solutions after solvent addition.

Observations show that DNA aqueous solutions do not mix with 1-butanol, and thus are insufficient for thin-film fabrication. Acetone consistently produces the least uniform films given extra discontinuities on the surface. Of the alcohols, isopropanol is the least able to consistently produce a uniform thin film. Methanol and ethanol perform similarly; however, methanol is more consistent in producing uniform films regardless of spin rate. UV–VIS analysis indicates that the differences in performance may be a result of how concentrations are changed after solvent addition. This may also be a result of conformational changes to DNA structure as a result of alcohol addition, and due to these changes, the final film uniformity may be greatly affected by alcohol choice [40]. Greater addition of isopropanol may induce greater conformational changes that are ill suited for fabrication of thin films. Importantly, it was found the alcohol-added solutions are generally more capable of producing more uniform films, and optimal uniformity will not always be at the highest spin rate.

## Figures and Tables

**Table 1:** Average thickness (nm) and standard deviation (nm) from 5 × 5 measurements of a pure water–DNA sample set 1.

Pure Water–DNA Sample Set 1	0.4 wt.%	0.8 wt.%	1.2 wt.%
1k RPM	Ave: 28.84 Std: 6.35 Std/Ave: 22.04%	Ave: 184.0 Std: 14.08 Std/Ave: 7.66%	Ave: 216.5 Std: 5.52 Std/Ave: 2.56%
3k RPM	Ave: 15.88 Std: 0.24 Std/Ave: 1.50%	Ave: 76.88 Std: 1.86 Std/Ave: 2.42%	Ave: 67.71 Std: 2.43 Std/Ave: 3.59%
5k RPM	Ave: 10.86 Std: 0.1 Std/Ave: 1.50%	Ave: 50.85 Std: 2.0 Std/Ave: 3.93%	Ave: 55.84 Std: 1.53 Std/Ave: 2.74%

**Table 2:** Average thickness (nm) and standard deviation (nm) from 5 × 5 measurements of a pure water–DNA sample set 2.

Pure Water DNA Sample Set 2	0.4 wt.%	0.8 wt.%	1.2 wt.%
1k RPM	Ave: 38.6 Std: 5.05 Std/Ave: 13.1%	Ave: 202.4 Std: 47.57 Std/Ave: 23.5%	Ave: 412.7 Std: 103.0 Std/Ave: 24.96%
3k RPM	Ave: 31.7 Std: 4.04 Std/Ave: 12.8%	Ave: 104.9 Std: 59.7 Std/Ave: 57.2%	Ave: 212.2 Std: 71.5 Std/Ave: 33.6%
5k RPM	Ave: 19.14 Std: 3.90 Std/Ave: 20.3%	Ave: 49.7 Std: 4.51 Std/Ave: 9.07%	Ave: 57.9 Std: 25.6 Std/Ave: 29.1%

**Table 3:** Average thickness (nm) and standard deviation (nm) from 5 × 5 measurements of 0.5:2 MeOH/H_2_O DNA solution. An amount of 2 mL of aqueous DNA with varying DNA concentration solutions diluted with 0.5 mL of methanol solvent.

0.5:2 (MeOH/H_2_O)	0.4 wt.% + 25% Volume of MeOH	0.8 wt.% + 25% Volume of MeOH	1.2 wt.% + 25% Volume of MeOH
1k RPM	Ave: 54.58 Std: 5.28 Std/Ave: 9.67%	Ave: 251.82 Std: 12.42 Std/Ave: 4.9%	Ave: 349.60 Std: 88.58 Std/Ave: 25.3%
3k RPM	Ave: 16.27 Std: 0.16 Std/Ave: 0.98%	Ave: 76.20 Std: 1.90 Std/Ave: 2.49%	Ave: 106.77 Std: 2.15 Std/Ave: 2.01%
5k RPM	Ave: 11.6 Std: 0.14 Std/Ave: 1.2%	Ave: 49.23 Std: 1.35 Std/Ave: 2.74%	Ave: 76.19 Std: 0.98 Std/Ave: 1.29%

**Table 4:** Average thickness (nm) and standard deviation (nm) from 5 × 5 measurements of 1:1 MeOH/H_2_O DNA solution. An amount of 2 mL of aqueous DNA with varying DNA concentrations diluted with 2 mL of methanol solvent.

1:1 (MeOH/H_2_O)	0.4 wt.% + 100% Volume of MeOH	0.8 wt.% + 100% Volume of MeOH	1.2 wt.% + 100% Volume of MeOH
1k RPM	Ave: 38.56 Std: 5.05 Std/Ave: 13.1%	Ave: 230.87 Std: 21.36 Std/Ave: 9.25%	Ave: 256.45 Std: 42.72 Std/Ave: 16.7%
3k RPM	Ave: 12.49 Std: 0.84 Std/Ave: 6.72%	Ave: 49.68 Std: 0.87 Std/Ave: 1.75%	Ave: 93.24 Std: 1.57 Std/Ave: 1.68%
5k RPM	Ave: 9.28 Std: 0.15 Std/Ave: 1.62%	Ave: 31.51 Std: 1.5 Std/Ave: 4.76%	Ave: 62.14 Std: 2.20 Std/Ave: 3.54%

**Table 5:** Average thickness (nm) and standard deviation (nm) from 5 × 5 measurements of 1:1 EtOH/H_2_O, 1:1 isopropanol/H_2_O, and 1:1 acetone/H_2_O DNA solutions. A total of 2 mL of aqueous DNA at 8 mg/mL (0.8 wt.%) diluted with 2 mL of ethanol, isopropanol, or acetone solvent.

1:1 (Solvent/DNA Solution)	0.8 wt.% + 100% Volume of Ethanol	0.8 wt.% + 100% Volume of Isopropanol	0.8 wt.% + 100% Volume of Acetone
1k RPM	Ave: 215.55 Std: 15.0 Std/Ave: 6.96%	Ave: 230.97 Std: 16.96 Std/Ave: 7.3%	Ave: 160.02 Std: 26.48 Std/Ave: 16.5%
3k RPM	Ave: 42.46 Std: 2.66 Std/Ave: 6.26%	Ave: 43.85 Std: 4.34 Std/Ave: 9.89%	Ave: 45.46 Std: 1.63 Std/Ave: 3.58%
5k RPM	Ave: 25.23 Std: 2.41 Std/Ave: 9.55%	Ave: 25.2 Std: 7.18 Std/Ave: 28.5%	Ave: 31.39 Std: 1.53 Std/Ave: 4.9%

## Data Availability

The data that support the findings of this study are available from the corresponding author upon reasonable request.
